# Convergence analysis of an iterative algorithm for the extended regularized nonconvex variational inequalities

**DOI:** 10.1186/s13660-017-1347-6

**Published:** 2017-04-24

**Authors:** Ying Zhao, Luoyi Shi, Rudong Chen

**Affiliations:** Department of Mathematics, Tianjin Polytechnic University, Tianjin, 300387 P.R. China

**Keywords:** general nonconvex variational inclusions, convergence analysis, fixed point problem

## Abstract

In this paper, we suggest and analyze a new system of extended regularized nonconvex variational inequalities and prove the equivalence between the aforesaid system and a fixed point problem. We introduce a new perturbed projection iterative algorithm with mixed errors to find the solution of the system of extended regularized nonconvex variational inequalities. Furthermore, under moderate assumptions, we research the convergence analysis of the suggested iterative algorithm.

## Introduction

Variational inequality was introduced and studied by Stampacchia [1] in 1964. It has been recognized as a suitable mathematical model to deal with many problems arising in different fields, such as optimization theory, game theory, partial differential equations, and economic equilibrium mechanics; see [2–4] and the references therein. Because of its importance and active impact in the nonlinear analysis and optimization, variational inequality has been explosively growing in both theory and applications; see, for example, [5–8]. Specially, one of the significant generalizations of variational inequality is the general variational inequality which was introduced and investigated by Noor [9]. Subsequently, Balooee *et al.* [10, 11] introduced an algorithm for solving the extended general mixed variational inequalities. However, most of the results related to the existence of solutions and iterative methods for variational inequality problems have been investigated and considered so far to the case where the underlying set is convex.

It is worth to mention that in many of the alluded applications, the set involved is not convex. To overcome the difficulty caused by the nonconvexity of the set, Clarke *et al.* [12] introduced a new class of nonconvex sets, that is, proximally smooth sets. Moreover, they were introduced by Poliquin *et al.* [13] but called the uniformly prox-regular sets. These kinds of sets are used in many nonconvex applications, such as differential inclusions, dynamical systems, and optimization; see [14, 15] and the references therein. It is well known that the uniformly prox-regular sets are nonconvex and include the convex sets as special cases. In 2009, Noor [16] considered a new class of variational inequalities, called the general nonconvex variational inequalities, and introduced the convergence analysis of the suggested iterative algorithms underlying the uniformly prox-regular sets. For more numerical methods for solving the variational inequalities and their generalizations in the context of nonconvexity, we refer the reader to [17–20] and the references therein.

The projection technique was introduced by Lions and Stampacchia [2]. It is one of the most widely used methods to study the variational inequalities, and this technique is devoted to establishing the equivalence between the variational inequalities and a fixed point problem which uses the concept of projection.

Inspired and motivated by the above works, in this paper, we introduce a new system of extended regularized nonconvex variational inequalities (SERNVI) and prove the equivalence between the SERNVI and a fixed point problem. Using this equivalent formulation, we consider a new perturbed projection iterative algorithm with mixed errors for finding the solution of the SERNVI. Under some moderate assumptions, we research the convergence analysis of the suggested iterative algorithm.

## Preliminaries

Let *H* be a real Hilbert space whose inner product and norm are denoted by $\langle\cdot,\cdot\rangle$ and $\|\cdot\|$, respectively. Let *K* be a nonempty closed subset of *H*, the usual distance function to the subset *K* is denoted by $d(\cdot,K)$, *i.e.*, $d(u,K)=\inf_{v\in{K}}\|u-v\|$. Now we recall the following basic definitions and results from nonsmooth analysis and nonlinear convex analysis.

### Definition 2.1

[21]

Let $u\in{H}$ be a point not lying in *K*. Let $v\in{K}$ be a point whose distance to *u* is minimal, i.e., $d(u,K)=\|u-v\|$, then *v* is called a closest point or a projection of *u* onto *K*. The set of all such closest points is denoted by $P_{K}$, that is,

$$P_{K}(u):=\bigl\{ v\in{K}:d(u,K)=\|u-v\|\bigr\} . $$

### Definition 2.2

[13]

The proximal normal cone of *K* at a point $u\in{K}$ is given by

$$N_{K}^{P}(u):=\bigl\{ \zeta\in{K}:\exists{\alpha}>0\mbox{ such that }u\in{P_{K}(u+\alpha \zeta)}\bigr\} . $$

Clarke *et al.* [21] gave the following characterization of the proximal normal cone $N_{K}^{P}(x)$.

### Lemma 2.3

*Let* *K* *be a nonempty closed subset in* *H*. *Then a vector* $\zeta\in {N_{K}^{P}(u)}$ *if and only if there exists a constant* $\alpha=\alpha(\zeta ,u)>0$ *such that*

2.1 $$ \langle\zeta,v-u\rangle\leq\alpha\|v-u\|^{2}, \quad \forall v \in{K}. $$

Inequality (2.1) is called the proximal normal inequality. Clarke *et al.* [21] considered the special case of the proximal normal cone $N_{K}^{P}(u)$, in which *K* is closed and convex; this case is an important one.

### Lemma 2.4

*Let* *K* *be a nonempty*, *closed and convex subset in* *H*. *Then* $\zeta\in {N_{K}^{P}(u)}$ *if and only if* $\langle\zeta,v-u\rangle\leq0$ *for all* $v\in{K}$.

### Definition 2.5

The Clarke normal cone of *C* at a point $u\in{K}$ is defined as

$$N_{K}^{C}(u)=\overline{\operatorname{co}} \bigl[N_{K}^{P}(u)\bigr], $$

where $\overline{\operatorname{co}}$ means the closure of the convex hull.

Clearly, $N_{K}^{P}(u)\subset{N_{K}^{C}(u)}$, but the converse is not true. $N_{K}^{P}(u)$ is always closed and convex, whereas $N_{K}^{C}(u)$ is convex, but may not be closed.

To overcome the difficulty caused by the nonconvexity of the set, Clarke *et al.* [12] introduced a new class of nonconvex sets, which are said to be proximally smooth sets. Subsequently, Poliquin *et al.* [13] considered the aforementioned set under the name of uniformly prox-regular sets. We take the following characterization verified in [12] as a definition of the uniformly prox-regular sets.

### Definition 2.6

For any $r\in(0,+\infty]$, a subset ${K_{r}}$ of *H* is said to be normalized uniformly prox-regular (or uniformly *r*-prox-regular) if every nonzero proximal normal to ${K_{r}}$ can be realized by an *r*-ball. This means that, for all $\bar{u}\in{K_{r}}$ and all $0\neq\zeta \in{N_{K}^{P}(\bar{u})}$ with $\|\zeta\|=1$,

$$\biggl\langle \frac{\zeta}{\|\zeta\|},u-\bar{u}\biggr\rangle \leq\frac{1}{2r}\|u- \bar {u}\|^{2},\quad \forall u\in{K_{r}}. $$

It is obvious that a closed subset of a Hilbert space is convex if and only if it is proximally smooth of radius $r>0$. In view of Definition 2.6, if $r=+\infty$, the uniform *r*-prox-regularity of $K_{r}$ is equivalent to the convexity of $K_{r}$. So, we set $K_{r}=K$, when $r=+\infty$. Moreover, a class of uniformly prox-regular sets is sufficiently large to include the class of convex sets, *P*-convex sets, $C^{1,1}$ submanifolds of *H*, the images under a $C^{1,1}$ diffeomorphism of convex sets and many other nonconvex sets.

We now recall the following well-known proposition which summarizes some significant consequences of the uniform prox-regularity. The proof of this result can be found in [12, 13].

### Proposition 2.7

*Let* $r\in{(0,\infty]}$ *and* $U(r)=\{u\in{H}:d(u,K_{r})< r\}$, *and let* $K_{r}$ *be a nonempty closed and uniformly* *r*-*prox*-*regular subset of* *H*. *Then the following results hold*:

(i) *For all* $x\in{K_{r}}$, *set* $P_{K_{r}}(x)\neq\emptyset$;
(ii) *For all* $r'\in{(0,r)}$, $P_{K_{r}}$ *is Lipschitz continuous with constant* $\frac{r}{r-r'}$ *on* $U(r')$;
(iii) *The proximal normal cone is closed as a set*-*valued mapping*.

## System of extended regularized nonconvex variational inequalities

In this section, we introduce a new system of extended regularized nonconvex variational inequalities and prove the equivalence between the aforesaid system and a fixed point problem.

Let $K_{r}$ be a uniformly *r*-prox-regular subset of *H*, and let $g_{i}:H\rightarrow{K_{r}}$, $h_{i}:H\rightarrow{H}$ ($i=1,\ldots,N$) be nonlinear single-valued mappings such that $g_{i}(H)\in{K_{r}}$. Let $T_{i}:H\times{H}\rightarrow{\operatorname{CB}(H)}$ ($i=1,\ldots,N$) ($\operatorname{CB}(H)$ means a nonempty closed and bounded subset of *H*) be nonlinear set-valued mappings, let $Q_{i}:H\rightarrow{H}$ ($i=1,\ldots,N$) be single-valued mappings. For any given constants $\rho_{i}>0$ ($i=1,\ldots,N$) and for all $x\in{H}$, $g_{i}(x)\in{K_{r}}$, the following problem of finding $x_{i}^{*}\in{H}$ ($i=1,\ldots,N$) with $h_{i}(x_{i}^{*})\in{K_{r}}$ ($i=1,\ldots,N$) and $u_{i}^{*}\in{T_{i}(x_{i+1}^{*},x_{i}^{*})}$ ($i=1,\ldots,N-1$), $u_{N}^{*}\in{T_{N}(x_{1}^{*},x_{N}^{*})}$ such that

3.1 $$ \textstyle\begin{cases} \langle\rho _{i}Q_{i}(u_{i}^{*})+h_{i}(x_{i}^{*})-g_{i}(x_{i+1}^{*}),g_{i}(x)-h_{i}(x_{i}^{*})\rangle+\frac {\lambda_{i}}{2r}\|g_{i}(x)-h_{i}(x_{i}^{*})\|^{2}\geq0 \\ \quad (i=1,\ldots,N-1), \\ \langle\rho_{N}Q_{N}(u_{N}^{*})+h_{N}(x_{N}^{*})-g_{N}(x_{1}^{*}),g_{N}(x)-h_{N}(x_{N}^{*})\rangle +\frac{\lambda_{N}}{2r}\|g_{N}(x)-h_{N}(x_{N}^{*})\|^{2}\geq0, \end{cases} $$

is called the system of extended regularized nonconvex variational inequalities (SERNVI), where $\lambda_{i}=\|\rho_{i}Q_{i}(u_{i}^{*})+h_{i}(x_{i}^{*})-g_{i}(x_{i+1}^{*})\|$ ($i=1,\ldots,N-1$), $\lambda_{N}=\|\rho_{N}Q_{N}(u_{N}^{*})+h_{N}(x_{N}^{*})-g_{N}(x_{1}^{*})\|$.

We note that if $i=1,2$, then problem (3.1) is equivalent to finding $(x_{1}^{*},x_{2}^{*})\in{H\times{H}}$ with $(h_{1}(x_{1}^{*}),h_{2}(x_{2}^{*}))\in{K_{r}\times{K_{r}}}$ and $u_{1}^{*}\in{T_{1}(x_{2}^{*},x_{1}^{*})}$, $u_{2}^{*}\in{T_{2}(x_{1}^{*},x_{2}^{*})}$ such that

3.2 $$ \textstyle\begin{cases} \langle\rho_{1}Q_{1}(u_{1}^{*})+h_{1}(x_{1}^{*})-g_{1}(x_{2}^{*}),g_{1}(x)-h_{1}(x_{1}^{*})\rangle +\frac{\lambda_{1}}{2r}\|g_{1}(x)-h_{1}(x_{1}^{*})\|^{2}\geq0, \\ \langle\rho_{2}Q_{2}(u_{2}^{*})+h_{2}(x_{2}^{*})-g_{2}(x_{1}^{*}),g_{2}(x)-h_{2}(x_{2}^{*})\rangle +\frac{\lambda_{2}}{2r}\|g_{2}(x)-h_{2}(x_{2}^{*})\|^{2}\geq0, \end{cases} $$

where $\lambda_{1}=\|\rho_{1}Q_{1}(u_{1}^{*})+h_{1}(x_{1}^{*})-g_{1}(x_{2}^{*})\|$, $\lambda_{2}=\|\rho_{2}Q_{2}(u_{2}^{*})+h_{2}(x_{2}^{*})-g_{2}(x_{1}^{*})\|$, which was introduced by Ansari *et al.* [18] in 2014.

If for each $i=1,2$, $g_{i}=h_{i}=g$, $Q_{i}\equiv{I}$, the identity operator $T_{i}=T:H\rightarrow{H}$ is an univariate nonlinear operator, then problem (3.1) reduces to the problem of finding $(x_{1}^{*},x_{2}^{*})\in{H\times{H}}$ such that

3.3 $$ \textstyle\begin{cases} \langle\rho_{1}T(x_{1}^{*})+g(x_{1}^{*})-g(x_{2}^{*}),g(x)-g(x_{1}^{*})\rangle+\frac {\lambda_{1}}{2r}\|g(x)-g(x_{1}^{*})\|^{2}\geq0, \\ \langle\rho_{2}T(x_{2}^{*})+g(x_{2}^{*})-g(x_{1}^{*}),g(x)-g(x_{2}^{*})\rangle+\frac {\lambda_{2}}{2r}\|g(x)-g(x_{2}^{*})\|^{2}\geq0, \end{cases} $$

where $\lambda_{1}=\|\rho_{1}T(x_{1}^{*})+g(x_{1}^{*})-g(x_{2}^{*})\|$, $\lambda_{2}=\|\rho_{2}T(x_{2}^{*})+g(x_{2}^{*})-g(x_{1}^{*})\|$. Problem (3.3) is called the system of general nonconvex variational inequalities and was introduced by Noor [19].

It is worth to mention that if $T_{i}=T:H\rightarrow{H}$ is an univariate nonlinear operator, $x_{i}^{*}=u$ ($i=1,\ldots,N$), $g_{i}=g$, $Q_{i}=h_{i}\equiv{I}$, the identity operator, then problem (3.1) changes into the problem of finding $u\in{K_{r}}$ such that

3.4 $$ \bigl\langle \rho{Tu}+u-g(u),g(v)-u\bigr\rangle +\frac{ \Vert \rho{Tu}+u-g(u) \Vert }{2r} \bigl\Vert g(v)-u \bigr\Vert ^{2}\geq0,\quad \forall{v\in{K_{r}}}, $$

which is called the general nonconvex variational inequality, introduced and investigated by Noor [16].

Moreover, if $g_{i}=h_{i}\equiv{I}$, the identity operator, then problem (3.4) is equivalent to finding $u\in{K_{r}}$ such that

3.5 $$ \langle\rho{Tu},v-u\rangle+\frac{\|\rho{Tu}\|}{2r}\|v-u\|^{2}\geq0,\quad \forall{v\in{K_{r}}}, $$

which is called the nonconvex variational inequality. For more details about the nonconvex variational inequality, we refer the reader to [17, 19] and the references therein.

We note that if $K_{r}\equiv{K}$, the convex set in *H*, then problem (3.5) is equivalent to finding $u\in{K}$ such that

3.6 $$ \langle{Tu,v-u}\rangle\geq0,\quad \forall{v\in{K}}. $$

An inequality of type (3.6), called the classical variational inequality, has been explosively growing in both theory and applications. For more formulation and numerical information about the methods, see [5–7].

We now establish the equivalence between the system of extended regularized nonconvex variational inequalities (3.1) and a system of nonconvex variational inclusions.

### Proposition 3.1

*Let* $Q_{i}$, $T_{i}$, $h_{i}$, $g_{i}$, *and* $\rho_{i}$ ($i=1,\ldots,N$) *be the same as system* (3.1) *such that* $g_{i}(H)\in{K_{r}}$ ($i=1,\ldots,N$). *Then* $x_{i}^{*}\in {H}$ ($i=1,\ldots,N$) *with* $h_{i}(x_{i}^{*})\in{K_{r}}$ *and* $u_{i}^{*}\in {T_{i}(x_{i+1}^{*},x_{i}^{*})}$ ($i=1,\ldots,N-1$), $u_{N}^{*}\in{T_{N}(x_{1}^{*},x_{N}^{*})}$ *is a solution of system* (3.1) *if and only if*

3.7 $$ \textstyle\begin{cases} g_{i}(x_{i+1}^{*})-h_{i}(x_{i}^{*})-\rho_{i}Q_{i}(u_{i}^{*})\in{N_{K_{r}}^{P}(h_{i}(x_{i}^{*}))}\quad (i=1,\ldots,N-1), \\ g_{N}(x_{1}^{*})-h_{N}(x_{N}^{*})-\rho_{N}Q_{N}(u_{N}^{*})\in{N_{K_{r}}^{P}(h_{N}(x_{N}^{*}))}, \end{cases} $$

*where* $N_{K_{r}}^{P}(s)$ *denotes the* *P*-*normal cone of* ${K_{r}}$ *at s in the sense of nonconvex analysis*.

### Proof

Let $x_{i}^{*}\in{H}$ with $h_{i}(x_{i}^{*})\in{K_{r}}$ ($i=1,\ldots,N$) and $u_{i}^{*}\in{T_{i}(x_{i+1}^{*},x_{i}^{*})}$ ($i=1,\ldots,N-1$), $u_{N}^{*}\in{T_{N}(x_{1}^{*},x_{N}^{*})}$ be a solution of system (3.1).

If $g_{i}(x_{i+1}^{*})-h_{i}(x_{i}^{*})-\rho_{i}Q_{i}(u_{i}^{*})=0$, since the vector zero always belongs to any normal cone, it follows that

$$g_{i}\bigl(x_{i+1}^{*}\bigr)-h_{i} \bigl(x_{i}^{*}\bigr)-\rho_{i}Q_{i} \bigl(u_{i}^{*}\bigr)\in{N_{K_{r}}^{P} \bigl(h_{i}\bigl(x_{i}^{*}\bigr)\bigr)}. $$

If $g_{i}(x_{i+1}^{*})-h_{i}(x_{i}^{*})-\rho_{i}Q_{i}(u_{i}^{*})\neq0$, for all $x\in{H}$, we observe that

3.8 $$ \bigl\langle {g_{i}\bigl(x_{i+1}^{*}\bigr)-h_{i} \bigl(x_{i}^{*}\bigr)-\rho _{i}Q_{i} \bigl(u_{i}^{*}\bigr),g_{i}(x)-h_{i} \bigl(x_{i}^{*}\bigr)}\bigr\rangle \leq\frac{\lambda_{i}}{2r} \bigl\Vert g_{i}(x)-h_{i}\bigl(x_{i}^{*}\bigr) \bigr\Vert ^{2}, $$

where $\lambda_{i}=\|\rho_{i}Q_{i}(u_{i}^{*})+h_{i}(x_{i}^{*})-g_{i}(x_{i+1}^{*})\|$ ($i=1,\ldots,N-1$). It follows from Lemma 2.3 and (3.8) that

$$g_{i}\bigl(x_{i+1}^{*}\bigr)-h_{i} \bigl(x_{i}^{*}\bigr)-\rho_{i}Q_{i} \bigl(u_{i}^{*}\bigr)\in {N_{K_{r}}^{P} \bigl(h_{i}\bigl(x_{i}^{*}\bigr)\bigr)}\quad (i=1,\ldots,N-1). $$

Similarly,

$$g_{N}\bigl(x_{1}^{*}\bigr)-h_{N} \bigl(x_{N}^{*}\bigr)-\rho_{N}Q_{N} \bigl(u_{N}^{*}\bigr)\in{N_{K_{r}}^{P} \bigl(h_{N}\bigl(x_{N}^{*}\bigr)\bigr)}. $$

On the contrary, if $x_{i}^{*}\in{H}$ with $h_{i}(x_{i}^{*})\in{K_{r}}$ ($i=1,\ldots,N$) and $u_{i}^{*}\in{T_{i}(x_{i+1}^{*},x_{i}^{*})}$ ($i=1,\ldots,N-1$), $u_{N}^{*}\in{T_{N}(x_{1}^{*},x_{N}^{*})}$ is a solution of system (3.7). Using Definition 2.6, we can get that $x_{i}^{*}\in{H}$ with $h_{i}(x_{i}^{*})\in{K_{r}}$ ($i=1,\ldots,N$) and $u_{i}^{*}\in{T_{i}(x_{i+1}^{*},x_{i}^{*})}$ ($i=1,\ldots,N-1$), $u_{N}^{*}\in{T_{N}(x_{1}^{*},x_{N}^{*})}$ is a solution of system (3.1). This completes the proof. □

Note that problem (3.7) is *called a system of general nonconvex variational inclusions* (SGNVI) *associated with a system of extended regularized nonconvex variational inequalities*.

Now, we establish the equivalence between SERNVI (3.1) and a fixed point problem, which is very useful for our analysis. It is worth to mention that several fixed point methods, such as (hybrid) projection algorithm and Mann iterative algorithm, have been developed for solving the nonlinear problem; see [22–24] and the references therein.

### Theorem 3.2

*Suppose that* $K_{r}$, $Q_{i}$, $T_{i}$, $h_{i}$, $g_{i}$, *and* $\rho_{i}$ ($i=1,\ldots,N$) *are the same as system* (3.1) *such that* $g_{i}(H)\in{K_{r}}$ ($i=1,\ldots,N$). *Then* $x_{i}^{*}\in{H}$ *with* $h_{i}(x_{i}^{*})\in{K_{r}}$ ($i=1,\ldots,N$) *and* $u_{i}^{*}\in{T_{i}(x_{i+1}^{*},x_{i}^{*})}$ ($i=1,\ldots,N-1$), $u_{N}^{*}\in {T_{N}(x_{1}^{*},x_{N}^{*})}$ *is a solution of system* (3.1) *if and only if*

3.9 $$ \textstyle\begin{cases} h_{i}(x_{i}^{*})=P_{K_{r}}[g_{i}(x_{i+1}^{*})-\rho_{i}Q_{i}(u_{i}^{*})]\quad (i=1,\ldots,N-1), \\ h_{N}(x_{N}^{*})=P_{K_{r}}[g_{N}(x_{1}^{*})-\rho_{N}Q_{N}(u_{N}^{*})], \end{cases} $$

*with* $\rho_{i}<\frac{r'}{1+\|Q_{i}(u_{i}^{*})\|}$ ($i=1,\ldots,N-1$), $\rho_{N}<\frac{r'}{1+\|Q_{N}(u_{N}^{*})\|}$, *for some* $r'\in(0,r)$, *where* $P_{K_{r}}$ *is the projection of* *H* *onto* $K_{r}$.

### Proof

Suppose $x_{i}^{*}\in{H}$ with $h_{i}(x_{i}^{*})\in{K_{r}}$ ($i=1,\ldots,N$) and $u_{i}^{*}\in{T_{i}(x_{i+1}^{*},x_{i}^{*})}$ ($i=1,\ldots,N-1$), $u_{N}^{*}\in{T_{N}(x_{1}^{*},x_{N}^{*})}$ is a solution of system (3.1). Since $g_{i}(x_{i}^{*})\in{K_{r}}$ ($i=1,\ldots,N$), and for some $r'\in(0,r)$, $\rho_{i}<\frac{r'}{1+\|Q_{i}(u_{i}^{*})\|}$ ($i=1,\ldots,N-1$), $\rho_{N}<\frac{r'}{1+\|Q_{N}(u_{N}^{*})\|}$, we can check that $g_{i}(x_{i+1}^{*})-\rho_{i}Q_{i}(u_{i}^{*})$ and $g_{N}(x_{1}^{*})-\rho_{N}Q_{N}(u_{N}^{*})$ belong to $U(r')$, which implies that equations (3.9) are well defined.

From Theorem 3.2 and the above mentioned works, we can get

$$\begin{aligned}& \textstyle\begin{cases} g_{i}(x_{i+1}^{*})-h_{i}(x_{i}^{*})-\rho_{i}Q_{i}(u_{i}^{*})\in{N_{K_{r}}^{P}(h_{i}(x_{i}^{*}))}\quad (i=1,\ldots,N-1), \\ g_{N}(x_{1}^{*})-h_{N}(x_{N}^{*})-\rho_{N}Q_{N}(u_{N}^{*})\in{N_{K_{r}}^{P}(h_{N}(x_{N}^{*}))} \end{cases}\displaystyle \\& \quad \Longleftrightarrow\quad \textstyle\begin{cases} g_{i}(x_{i+1}^{*})-\rho_{i}Q_{i}(u_{i}^{*})\in{(I+N_{K_{r}}^{P})(h_{i}(x_{i}^{*}))}\quad (i=1,\ldots,N-1), \\ g_{N}(x_{1}^{*})-\rho_{N}Q_{N}(u_{N}^{*})\in{(I+N_{K_{r}}^{P})(h_{N}(x_{N}^{*}))} \end{cases}\displaystyle \\& \quad \Longleftrightarrow\quad \textstyle\begin{cases} h_{i}(x_{i}^{*})=P_{K_{r}}[g_{i}(x_{i+1}^{*})-\rho_{i}Q_{i}(u_{i}^{*})] \quad (i=1,\ldots,N-1), \\ h_{N}(x_{N}^{*})=P_{K_{r}}[g_{N}(x_{1}^{*})-\rho_{N}Q_{N}(u_{N}^{*})], \end{cases}\displaystyle \end{aligned}$$

where *I* is an identity mapping and $P_{K_{r}}=(I+N_{K_{r}}^{P})^{-1}$. This completes the proof. □

## Main result

In this section, we use the foregoing equivalent alternative formulation (3.9) to consider new perturbed projection iterative algorithms with mixed errors for finding the solution of SERNVI (3.1). For convenience, we now recall some definitions, and they will be powerful tools in our analysis.

### Definition 4.1

An operator $g:H\rightarrow{H}$ is called

(i) monotone if
  $$\bigl\langle {g(x)-g(y),x-y}\bigr\rangle \geq0,\quad \forall{x,y\in{H}}; $$
(ii) strongly monotone if there exists a constant $\eta>0$ such that
  $$\bigl\langle {g(x)-g(y),x-y}\bigr\rangle \geq{\eta\|x-y\|^{2}},\quad \forall{x,y\in{H}}; $$
(iii) Lipschitz continuous if there exists a constant $\sigma>0$ such that
  $$\bigl\Vert g(x)-g(y) \bigr\Vert \leq\sigma \Vert x-y \Vert ,\quad \forall{x,y\in{H}}. $$

### Definition 4.2

Let $T:H\times{H}\rightarrow{2^{H}}$ be a set-valued mapping, a nonlinear single-valued mapping $Q:H\rightarrow{H}$ is called

(i) monotone if for any $u\in{T(x,y)}$, $v\in{T(y,x)}$ such that
  $$\bigl\langle {Q(u)-Q(v),x-y}\bigr\rangle \geq0,\quad \forall{x,y\in{H}}; $$
(ii) relaxed $(\kappa,\lambda)$ cocoercive if for any $u\in{T(x,y)}$, $v\in{T(y,x)}$, there exist constants $\kappa>0$ and $\lambda>0$ such that
  $$\bigl\langle {Q(u)-Q(v),x-y}\bigr\rangle \geq{-\kappa \bigl\Vert Q(u)-Q(v) \bigr\Vert ^{2}+\lambda \Vert x-y \Vert ^{2}},\quad \forall{x,y\in{H}}; $$
(iii) *μ*-Lipschitz continuous if there exists a constant $\mu>0$ such that
  $$\bigl\Vert Q(x_{1})-Q(x_{2}) \bigr\Vert \geq\mu \Vert x_{1}-x_{2} \Vert ,\quad \forall{x_{1},x_{2} \in{H}}. $$

Note that the notion of the cocoercivity is applied in several directions, especially to solving the variational inequality problems using the auxiliary problem principle and the projection methods, see [25]; while the notion of the relaxed cocoercivity is more general than the strong monotonicity as well as cocoercivity. For more details about the relaxed cocoercive variational inequalities and variational inclusions, see, for example, [26].

### Definition 4.3

A two-variable set-valued mapping $T:H\times{H}\rightarrow{2^{H}}$ is said to be *π*-*D̂*-Lipschitz continuous in the first variable if for all $x,x',y,y'\in{H}$, there exists a constant $\pi>0$ such that

$$\widehat{D}\bigl(T(x,y),T\bigl(x',y'\bigr)\bigr)\leq \pi \bigl\Vert x-x' \bigr\Vert , $$

where *D̂* is the Hausdorff pseudo-metric, that is, for any two nonempty subsets *A* and *B* of *H*,

$$\widehat{D}(A,B)=\max\Bigl\{ \sup_{x\in{A}}d(x,B),\sup _{y\in{B}}d(y,A)\Bigr\} . $$

In what follows, we introduce a new perturbed projection iterative algorithm with mixed errors to find the solution of SERNVI (3.1). For convenience, we assume that $K_{r}$ is a uniformly *r*-prox-regular subset of *H* with $r>0$, also let $r'\in(0,r)$ and set $\delta=\frac{r}{r-r'}$.

### Algorithm 4.4

Suppose $Q_{i}$, $T_{i}$, $h_{i}$, $g_{i}$, and $\rho_{i}$ ($i=1,\ldots,N$) are the same as system (3.1) such that $g_{i}(H)\in{K_{r}}$ ($i=1,\ldots,N$), the iterative sequence $\{(x_{1}^{n},\ldots,x_{N}^{n})\}_{n=0}^{\infty}$ in ${\underbrace{ H\times\cdots\times{H} } _{N\text{-times}} }$ for an arbitrarily chosen initial point $(x_{1}^{0},\ldots,x_{N}^{0})\in{\underbrace{ H\times\cdots\times{H} } _{N\text{-times}} }$ is given as follows:

4.1 $$ \textstyle\begin{cases} x_{i}^{n+1}=(1-\alpha_{n})x_{i}^{n}+\alpha _{n}(x_{i}^{n}-h_{i}(x_{i}^{n})+P_{k_{r}}(g_{i}(x_{i+1}^{n})-\rho_{i}Q_{i}(u_{i}^{n}))) \\ \hphantom{x_{i}^{n+1}={}}{}+\alpha_{n}e_{i}^{n}+q_{i}^{n}\quad (i=1,\ldots,N-1), \\ x_{N}^{n+1}=(1-\alpha_{n})x_{N}^{n}+\alpha _{n}(x_{N}^{n}-h_{N}(x_{N}^{n})+P_{k_{r}}(g_{N}(x_{1}^{n})-\rho_{N}Q_{N}(u_{N}^{n}))) \\ \hphantom{x_{N}^{n+1}={}}{}+\alpha_{n}e_{N}^{n}+q_{N}^{n}, \end{cases} $$

where $\{\alpha_{n}\}_{n=0}^{\infty}$ is a sequence in $[0,1]$ such that $\sum_{n=0}^{\infty}{\alpha_{n}}=\infty$, and $\{e_{i}^{n}\}_{n=0}^{\infty}$, $\{q_{i}^{n}\}_{n=0}^{\infty}$ ($i=1,\ldots,N$) are sequences in *H* to consider a possible inexact point of the resolvent operator satisfying the following conditions:

4.2 $$ \textstyle\begin{cases} e_{i}^{n}=e_{i'}^{n}+e_{i''}^{n} \quad (i=1,\ldots,N), \\ \lim_{n\rightarrow\infty}\|e_{1'}^{n},\ldots,e_{N'}^{n}\|_{*}=0,\qquad \sum_{n=0}^{\infty}\|e_{1''}^{n},\ldots,e_{N''}^{n}\|_{*}< \infty, \\ \sum_{n=0}^{\infty}\|q_{1}^{n},\ldots,q_{N}^{n}\|_{*}< \infty. \end{cases} $$

Moreover, by Nadler theorem [27], there exist

$$\textstyle\begin{cases} u_{i}^{n}\in{T_{i}^{n}(x_{i+1}^{n},x_{i}^{n})}\quad (i=1,\ldots,N-1), \\ \|u_{i}^{n}-u_{i}^{n+1}\|\leq(1+\frac{1}{i})\widehat {D}(T_{i}(x_{i+1}^{n},x_{i}^{n}),T_{i}(x_{i+1}^{n+1},x_{i}^{n+1})), \\ u_{N}^{n}\in{T_{N}(x_{1}^{n},x_{N}^{n})}, \\ \|u_{N}^{n}-u_{N}^{n+1}\|\leq(1+\frac{1}{N})\widehat {D}(T_{N}(x_{1}^{n},x_{N}^{n}),T_{N}(x_{1}^{n+1},x_{N}^{n+1})). \end{cases} $$

Before establishing the convergence analysis of the aforesaid algorithm, we review the well-known property, which provides the main mathematical results of this section.

### Lemma 4.5

[28]

*Let* ${a_{n}}$, ${b_{n}}$ *and* ${c_{n}}$ *be three nonnegative real sequences satisfying the following condition*. *There exists a natural number* $n_{0}$ *such that*

$$a_{n+1}\leq(1-t_{n})a_{n}+b_{n}t_{n}+c_{n}, \quad \forall n\geq{n_{0}}, $$

*where* $t_{n}\in[0,1]$, $\sum_{n=0}^{\infty}{t_{n}}=\infty$, $\lim_{n\rightarrow\infty}b_{n}=0$ *and* $\sum_{n=0}^{\infty}{c_{n}}<\infty$. *Then* $\lim_{n\rightarrow\infty}a_{n}=0$.

Next, the convergence of the iterative sequence generated by Algorithm 4.4 to a solution of SERNVI (3.1) is demonstrated.

### Theorem 4.6

*Suppose* $Q_{i}$, $T_{i}$, $h_{i}$, $g_{i}$, *and* $\rho_{i}$ ($i=1,\ldots,N$) *are the same as system* (3.1) *such that* $g_{i}(H)\in{K_{r}}$ ($i=1,\ldots,N$). *Furthermore*, *for each* $i=1,\ldots,N$,

(i) $Q_{i}$ *is* $(\kappa_{i},\lambda_{i})$-*relaxed cocoercive with constants* $\kappa_{i}>0$, $\lambda_{i}>0$;
(ii) $Q_{i}$ *is* $\mu_{i}$-*Lipschitz continuous with a constant* $\mu_{i}>0$;
(iii) $T_{i}$ *is* $\pi_{i}-\widehat{D}$-*Lipschitz continuous in the first variable with a constant* $\pi_{i}>0$;
(iv) $g_{i}$ *is* $\eta_{i}$-*strongly monotone and* $\sigma_{i}$-*Lipschitzian with constants* $\eta_{i}>0$, $\sigma_{i}>0$;
(v) $h_{i}$ *is* $\xi_{i}$-*strongly monotone and* $\zeta_{i}$-*Lipschitzian with constants* $\xi_{i}>0$, $\zeta_{i}>0$.

*If the constants* $\rho_{i}$ ($i=1,\ldots,N$) *satisfy the following conditions*:

4.3 $$ \rho_{i}< \frac{r'}{1+\|Q_{i}(u_{i})\|}, $$

*for some* $r'\in{(0,r)}$ *and*

4.4 $$ \textstyle\begin{cases} |\rho_{i}-\frac{\lambda_{i}-\kappa_{i}\mu_{i}^{2}(1+\frac{1}{i})^{2}\pi_{i}^{2}}{\mu _{i}^{2}(1+\frac{1}{i})^{2}\pi_{i}^{2}}| \\ \quad < \frac{\sqrt{\delta^{2}(\lambda_{i}-\kappa_{i}\mu_{i}^{2}(1+\frac{1}{i})^{2}\pi _{i}^{2})^{2}-\mu_{i}^{2}(1+\frac{1}{i})^{2}\pi_{i}^{2}\{\delta^{2}-(1-\theta_{i+1}-\delta \sqrt{1-2\eta_{i}+\sigma_{i}^{2}})^{2}\}}}{\delta\mu_{i}^{2}(1+\frac{1}{i})^{2}\pi _{i}^{2}}\quad (i=1,\ldots,N-1), \\ |\rho_{N}-\frac{\lambda_{N}-\kappa_{N}\mu_{N}^{2}(1+\frac{1}{N})^{2}\pi_{N}^{2}}{\mu _{N}^{2}(1+\frac{1}{N})^{2}\pi_{N}^{2}}| \\ \quad < \frac{\sqrt{\delta^{2}(\lambda_{N}-\kappa_{N}\mu_{N}^{2}(1+\frac{1}{N})^{2}\pi _{N}^{2})^{2}-\mu_{N}^{2}(1+\frac{1}{N})^{2}\pi_{N}^{2}\{\delta^{2}-(1-\theta_{1}-\delta \sqrt{1-2\eta_{N}+\sigma_{N}^{2}})^{2}\}}}{\delta\mu_{N}^{2}(1+\frac{1}{N})^{2}\pi _{N}^{2}}, \\ \delta(\lambda_{i}-\kappa_{i}\mu_{i}^{2}(1+\frac{1}{i})^{2}\pi_{i}^{2}) \\ \quad >\mu_{i}(1+\frac{1}{i})\pi_{i}\sqrt{\delta^{2}-(1-\theta_{i+1}-\delta\sqrt {1-2\eta_{i}+\sigma_{i}^{2}})^{2}}\quad (i=1,\ldots,N-1), \\ \delta(\lambda_{N}-\kappa_{N}\mu_{N}^{2}(1+\frac{1}{N})^{2}\pi_{N}^{2})>\mu_{N}(1+\frac {1}{N})\pi_{N}\sqrt{\delta^{2}-(1-\theta_{1}-\delta\sqrt{1-2\eta_{N}+\sigma _{N}^{2}})^{2}}, \\ \delta>1-\theta_{i+1}-\delta\sqrt{1-2\eta_{i}+\sigma_{i}^{2}}\quad (i=1,\ldots ,N-1), \\ \delta>1-\theta_{1}-\delta\sqrt{1-2\eta_{N}+\sigma_{N}^{2}}, \\ \theta_{i}=\sqrt{1-2\xi_{i}+\zeta_{i}^{2}}\quad (i=1,\ldots,N), \\ 2\xi_{i}< 1+\zeta_{i}^{2}\quad (i=1,\ldots,N). \end{cases} $$

*Then the iterative sequence* $\{x_{i}^{n}\}_{n=0}^{\infty}$ ($i=1,\ldots,N$) *generated by Algorithm* 4.4 *strongly converges to* $(x_{1}^{*},\ldots,x_{N}^{*})$.

### Proof

Since $g_{i}(x_{i}^{*})\in{K_{r}}$ ($i=1,\ldots,N$), $\rho_{i}<\frac{r'}{1+\|Q_{i}(u_{i}^{*})\|}$ ($i=1,\ldots,N-1$) and $\rho_{N}<\frac{r'}{1+\|Q_{N}(u_{N}^{*})\|}$, it follows from Theorem 3.2 that $x_{i}^{*}\in{H}$ satisfies equation (3.9). Moreover, since the solution of the system of extended regularized nonconvex variational inequalities is a singleton set, hence, for each positive integer *n*, we obtain

4.5 $$ \textstyle\begin{cases} x_{i}^{*}=(1-\alpha_{n})x_{i}^{*}+\alpha _{n}(x_{i}^{*}-h_{i}(x_{i}^{*})+P_{k_{r}}(g_{i}(x_{i+1}^{*})-\rho_{i}Q_{i}(u_{i}^{*}))) \\ \quad (i=1,\ldots,N), \\ x_{N}^{*}=(1-\alpha_{n})x_{N}^{*}+\alpha _{n}(x_{N}^{*}-h_{N}(x_{N}^{*})+P_{k_{r}}(g_{N}(x_{1}^{*})-\rho_{N}Q_{N}(u_{N}^{*}))), \end{cases} $$

where the sequence $\{\alpha_{n}\}$ is the same as in Algorithm 4.4.

Let $x_{i}^{n+1},{x}_{i}^{*}\in{H}$ be given, by Proposition 2.7, (4.1) and (4.5), we observe that

4.6 $$\begin{aligned}& \bigl\Vert x_{i}^{n+1}-x_{i}^{*} \bigr\Vert \\& \quad \leq (1-\alpha_{n}) \bigl\Vert x_{i}^{n}-x_{i}^{*} \bigr\Vert +\alpha_{n} \bigl\Vert \bigl\{ x_{i}^{n}-h_{i} \bigl(x_{i}^{n}\bigr)+P_{k_{r}}\bigl(g_{i} \bigl(x_{i+1}^{n}\bigr)-\rho_{i}Q_{i} \bigl(u_{i}^{n}\bigr)\bigr)\bigr\} \\& \qquad {}-\bigl\{ x_{i}^{*}-h_{i}\bigl(x_{i}^{*} \bigr)+P_{k_{r}}\bigl(g_{i}\bigl(x_{i+1}^{*}\bigr)- \rho_{i}Q_{i}\bigl(u_{i}^{*}\bigr)\bigr)\bigr\} \bigr\Vert +\alpha_{n} \bigl\Vert e_{i}^{n} \bigr\Vert + \bigl\Vert q_{i}^{n} \bigr\Vert \\& \quad \leq (1-\alpha_{n}) \bigl\Vert x_{i}^{n}-x_{i}^{*} \bigr\Vert +\alpha_{n}\bigl( \bigl\Vert x_{i}^{n}-x_{i}^{*}- \bigl(h_{i}\bigl(x_{i}^{n}\bigr)-h_{i} \bigl(x_{i}^{*}\bigr)\bigr) \bigr\Vert \\& \qquad {} +\delta \bigl\Vert g_{i}\bigl(x_{i+1}^{n}\bigr)-g_{i}\bigl(x_{i+1}^{*}\bigr)-\bigl( \rho_{i}Q_{i}\bigl(u_{i}^{n}\bigr)- \rho_{i}Q_{i}\bigl(u_{i}^{*}\bigr)\bigr) \bigr\Vert \bigr)+\alpha_{n} \bigl\Vert e_{i}^{n} \bigr\Vert + \bigl\Vert q_{i}^{n} \bigr\Vert \\& \quad \leq (1-\alpha_{n}) \bigl\Vert x_{i}^{n}-x_{i}^{*} \bigr\Vert +\alpha_{n}\bigl( \bigl\Vert x_{i}^{n}-x_{i}^{*}- \bigl(h_{i}\bigl(x_{i}^{n}\bigr)-h_{i} \bigl(x_{i}^{*}\bigr)\bigr) \bigr\Vert \\& \qquad {}+\delta\bigl\{ \bigl\Vert x_{i+1}^{n}-x_{i+1}^{*}-\bigl(g_{i}\bigl(x_{i+1}^{n} \bigr)-g_{i}\bigl(x_{i+1}^{*}\bigr)\bigr) \bigr\Vert + \bigl\Vert x_{i+1}^{n}-x_{i+1}^{*}-\rho_{i} \bigl(Q_{i}\bigl(u_{i}^{n}\bigr)-Q_{i} \bigl(u_{i}^{*}\bigr)\bigr) \bigr\Vert \bigr\} \bigr) \\& \qquad {}+\alpha_{n}\bigl( \bigl\Vert e_{i'}^{n} \bigr\Vert + \bigl\Vert e_{i''}^{n} \bigr\Vert \bigr)+ \bigl\Vert q_{i}^{n} \bigr\Vert . \end{aligned}$$

From the fact that $h_{i}$ is $\xi_{i}$-strongly monotone and $\zeta_{i}$-Lipschitzian, we can get that

4.7 $$\begin{aligned}& \bigl\Vert x_{i}^{n}-{x}_{i}^{*}- \bigl(h_{i}\bigl(x_{i}^{n}\bigr)-h_{i} \bigl({x}_{i}^{*}\bigr)\bigr) \bigr\Vert ^{2} \\& \quad = \bigl\Vert x_{i}^{n}-{x}_{i}^{*} \bigr\Vert ^{2}-2\bigl\langle {h_{i}\bigl(x_{i}^{n} \bigr)-h_{i}\bigl({x}_{i}^{*}\bigr),x_{i}^{n}-{x}_{i}^{*}} \bigr\rangle + \bigl\Vert h_{i}\bigl(x_{i}^{n} \bigr)-h_{i}\bigl({x}_{i}^{*}\bigr) \bigr\Vert ^{2} \\& \quad \leq \bigl\Vert x_{i}^{n}-{x}_{i}^{*} \bigr\Vert ^{2}-2\xi_{i} \bigl\Vert x_{i}^{n}-{x}_{i}^{*} \bigr\Vert ^{2}+\zeta_{i}^{2} \bigl\Vert x_{i}^{n}-x_{i}^{*} \bigr\Vert ^{2} \\& \quad = \bigl(1-2\xi_{i}+\zeta_{i}^{2}\bigr) \bigl\Vert x_{i}^{n}-{x}_{i}^{*} \bigr\Vert ^{2}. \end{aligned}$$

Using the fact that $g_{i}$ is $\eta_{i}$-strongly monotone and $\sigma_{i}$-Lipschitzian, we obtain

4.8 $$\begin{aligned}& \bigl\Vert x_{i+1}^{n}-{x}_{i+1}^{*}- \bigl(g_{i}\bigl(x_{i+1}^{n}\bigr)-g_{i} \bigl({x}_{i+1}^{*}\bigr)\bigr) \bigr\Vert ^{2} \\& \quad = \bigl\Vert x_{i+1}^{n}-{x}_{i+1}^{*} \bigr\Vert ^{2}-2\bigl\langle {g_{i}\bigl(x_{i+1}^{n} \bigr)-g_{i}\bigl({x}_{i+1}^{*}\bigr),x_{i+1}^{n}-{x}_{i+1}^{*}} \bigr\rangle + \bigl\Vert g_{i}\bigl(x_{i+1}^{n} \bigr)-g_{i}\bigl({x}_{i+1}^{*}\bigr) \bigr\Vert ^{2} \\& \quad \leq \bigl(1-2\eta_{i}+\sigma_{i}^{2} \bigr) \bigl\Vert x_{i+1}^{n}-{x}_{i+1}^{*} \bigr\Vert ^{2}. \end{aligned}$$

Since $Q_{i}$ is $\mu_{i}$-Lipschitz continuous and $T_{i}$ is $\pi _{i}-\widehat{D}$-Lipschitz continuous in the first variable, it follows that

4.9 $$\begin{aligned} \bigl\Vert Q_{i}\bigl(u_{i}^{n} \bigr)-Q_{i}\bigl({u}_{i}^{*}\bigr) \bigr\Vert \leq& \mu_{i} \bigl\Vert u_{i}^{n}-{u}_{i}^{*} \bigr\Vert \\ \leq&\mu_{i}\biggl(1+\frac{1}{i}\biggr)\widehat {D} \bigl(T_{i}\bigl(x_{i+1}^{n},x_{i}^{n} \bigr),T_{i}\bigl({x}_{i+1}^{*},{x}_{i}^{*}\bigr)\bigr) \\ \leq&\mu_{i}\biggl(1+\frac{1}{i}\biggr)\pi_{i} \bigl\Vert x_{i+1}^{n}-{x}_{i+1}^{*} \bigr\Vert . \end{aligned}$$

From (4.9) and $Q_{i}$ is $(\kappa_{i},\lambda_{i})$-relaxed cocoercive with respect to $Q_{i}$ and $T_{i}$, it is easy to see that

4.10 $$\begin{aligned}& \bigl\Vert x_{i+1}^{n}-{x}_{i+1}^{*}- \rho_{i}\bigl(Q_{i}\bigl(u_{i}^{n} \bigr)-Q_{i}\bigl({u}_{i}^{*}\bigr)\bigr) \bigr\Vert ^{2} \\& \quad = \bigl\Vert x_{i+1}^{n}-{x}_{i+1}^{*} \bigr\Vert ^{2}-2\rho_{i}\bigl\langle {Q_{i} \bigl(u_{i}^{n}\bigr)-Q_{i}\bigl({u}_{i}^{*} \bigr),x_{i+1}^{n}-{x}_{i+1}^{*}}\bigr\rangle \\& \qquad {}+\rho_{i}^{2} \bigl\Vert Q_{i} \bigl(u_{i}^{n}\bigr)-Q_{i}\bigl({u}_{i}^{*} \bigr) \bigr\Vert ^{2} \\& \quad \leq \bigl\Vert x_{i+1}^{n}-{x}_{i+1}^{*} \bigr\Vert ^{2}-2\rho_{i}\bigl(-\kappa_{i} \bigl\Vert Q_{i}\bigl(u_{i}^{n}\bigr)-Q_{i} \bigl({u}_{i}^{*}\bigr) \bigr\Vert ^{2} \\& \qquad {}+\lambda_{i} \bigl\Vert x_{i+1}^{n}-{x}_{i+1}^{*} \bigr\Vert ^{2}\bigr)+\rho_{i}^{2} \mu_{i}^{2} \bigl\Vert u_{i}^{n}-{u}_{i}^{*} \bigr\Vert ^{2} \\& \quad \leq \bigl\Vert x_{i+1}^{n}-{x}_{i+1}^{*} \bigr\Vert ^{2}-2\rho_{i}\biggl(-\kappa_{i} \mu_{i}^{2}\biggl(1+\frac {1}{i}\biggr)^{2} \pi_{i}^{2} \bigl\Vert x_{i+1}^{n}-{x}_{i+1}^{*} \bigr\Vert ^{2} \\& \qquad {}+\lambda_{i} \bigl\Vert x_{i+1}^{n}-{x}_{i+1}^{*} \bigr\Vert ^{2}\biggr)+\rho_{i}^{2} \mu_{i}^{2}\biggl(1+\frac {1}{i}\biggr)^{2} \pi_{i}^{2} \bigl\Vert x_{i+1}^{n}-{x}_{i+1}^{*} \bigr\Vert ^{2} \\& \quad = \biggl(1-2\rho_{i}\biggl(\lambda_{i}- \kappa_{i}\mu_{i}^{2}\biggl(1+\frac{1}{i} \biggr)^{2}\pi_{i}^{2}\biggr)+\rho _{i}^{2}\mu_{i}^{2}\biggl(1+ \frac{1}{i}\biggr)^{2}\pi_{i}^{2}\biggr) \bigl\Vert x_{i+1}^{n}-{x}_{i+1}^{*} \bigr\Vert ^{2}. \end{aligned}$$

Substituting (4.7), (4.8) and (4.10) in (4.6), we deduce that

4.11 $$\begin{aligned}& \bigl\Vert x_{i}^{n+1}-x_{i}^{*} \bigr\Vert \\& \quad \leq (1-\alpha_{n}) \bigl\Vert x_{i}^{n}-x_{i}^{*} \bigr\Vert +\alpha_{n}\bigl(\theta_{i} \bigl\Vert x_{i}^{n}-x_{i}^{*} \bigr\Vert +\phi _{i} \bigl\Vert x_{i+1}^{n}-x_{i+1}^{*} \bigr\Vert \bigr) \\& \qquad {}+\alpha_{n} \bigl\Vert e_{i'}^{n} \bigr\Vert + \bigl\Vert e_{i''}^{n} \bigr\Vert + \bigl\Vert q_{i}^{n} \bigr\Vert \\& \quad = \bigl(1-\alpha_{n}(1-\theta_{i})\bigr) \bigl\Vert x_{i}^{n}-x_{i}^{*} \bigr\Vert + \alpha_{n}\phi_{i} \bigl\Vert x_{i+1}^{n}-x_{i+1}^{*} \bigr\Vert \\& \qquad {} +\alpha_{n} \bigl\Vert e_{i'}^{n} \bigr\Vert + \bigl\Vert e_{i''}^{n} \bigr\Vert + \bigl\Vert q_{i}^{n} \bigr\Vert , \end{aligned}$$

where $\phi_{i}=\delta\{\sqrt{(1-2\eta_{i}+\sigma_{i}^{2})}+\sqrt{(1-2\rho_{i}(\lambda _{i}-\kappa_{i}\mu_{i}^{2}(1+\frac{1}{i})^{2}\pi_{i}^{2})+\rho_{i}^{2}\mu_{i}^{2}(1+\frac {1}{i})^{2}\pi_{i}^{2})}\}$, and $\theta_{i}=\sqrt{(1-2\xi_{i}+\zeta_{i}^{2})}$.

Now we define $\|\cdot\|_{*}$ on $\underbrace{ H\times\cdots\times{H} } _{N\text{-times}} $ as follows:

$$\bigl\Vert (x_{1},\ldots,x_{N}) \bigr\Vert _{*}= \Vert x_{1} \Vert +\cdots+ \Vert x_{N} \Vert ,\quad \mbox{for all }(x_{1},\ldots ,x_{N})\in\underbrace{ H\times\cdots \times{H} } _{N\text{-times}} . $$

It is obvious that $(\underbrace{ H\times\cdots\times{H} } _{N\text{-times}} ,\|\cdot\|_{*})$ is a Banach space. We derive from the definition of $\|\cdot\|_{*}$ and (4.11) that

4.12 $$\begin{aligned}& \bigl\Vert \bigl(x_{1}^{n+1},x_{2}^{n+1}, \ldots,x_{N}^{n+1}\bigr)-\bigl(x_{1}^{*},x_{2}^{*}, \ldots,x_{N}^{*}\bigr) \bigr\Vert _{*} \\& \quad \leq \bigl(1-\alpha_{n}(1-\theta_{1})\bigr) \bigl\Vert x_{1}^{n}-x_{1}^{*} \bigr\Vert + \alpha_{n}\phi_{1} \bigl\Vert x_{2}^{n}-x_{2}^{*} \bigr\Vert +\alpha_{n} \bigl\Vert e_{1'}^{n} \bigr\Vert \\& \qquad {}+ \bigl\Vert e_{1''}^{n} \bigr\Vert + \bigl\Vert q_{1}^{n} \bigr\Vert +\bigl(1-\alpha_{n}(1- \theta_{2})\bigr) \bigl\Vert x_{2}^{n}-x_{2}^{*} \bigr\Vert +\alpha_{n}\phi_{2} \bigl\Vert x_{3}^{n}-x_{3}^{*} \bigr\Vert \\& \qquad {}+\alpha_{n} \bigl\Vert e_{2'}^{n} \bigr\Vert + \bigl\Vert e_{2''}^{n} \bigr\Vert + \bigl\Vert q_{2}^{n} \bigr\Vert +\cdots+\bigl(1-\alpha _{n}(1-\theta_{N})\bigr) \bigl\Vert x_{N}^{n}-x_{N}^{*} \bigr\Vert \\& \qquad {}+\alpha_{n}\phi_{N} \bigl\Vert x_{1}^{n}-x_{1}^{*} \bigr\Vert + \alpha_{n} \bigl\Vert e_{N'}^{n} \bigr\Vert + \bigl\Vert e_{N''}^{n} \bigr\Vert + \bigl\Vert q_{N}^{n} \bigr\Vert \\& \quad \leq \bigl(1-\alpha_{n}(1-\vartheta)\bigr) \bigl\Vert \bigl(x_{1}^{n},x_{2}^{n},\ldots ,x_{N}^{n}\bigr)-\bigl(x_{1}^{*},x_{2}^{*}, \ldots,x_{N}^{*}\bigr) \bigr\Vert _{*} \\& \qquad {}+\alpha_{n} \bigl\Vert \bigl(e_{1'}^{n}, \ldots,e_{N'}^{n}\bigr) \bigr\Vert _{*}+ \bigl\Vert \bigl(e_{1''}^{n},\ldots ,e_{N''}^{n}\bigr) \bigr\Vert _{*}+ \bigl\Vert \bigl(q_{1}^{n}, \ldots,q_{N}^{n}\bigr) \bigr\Vert _{*} \\& \quad \leq \bigl(1-\alpha_{n}(1-\vartheta)\bigr) \bigl\Vert \bigl(x_{1}^{n},x_{2}^{n},\ldots ,x_{N}^{n}\bigr)-\bigl(x_{1}^{*},x_{2}^{*}, \ldots,x_{N}^{*}\bigr) \bigr\Vert _{*} \\& \qquad {} +\alpha_{n}(1-\vartheta)\frac{ \Vert (e_{1'}^{n},\ldots,e_{N'}^{n}) \Vert _{*}}{1-\vartheta}+ \bigl\Vert \bigl(e_{1''}^{n},\ldots,e_{N''}^{n}\bigr) \bigr\Vert _{*}+ \bigl\Vert \bigl(q_{1}^{n},\ldots ,q_{N}^{n}\bigr) \bigr\Vert _{*}, \end{aligned}$$

where $\vartheta=\Theta+\Phi$, $\Theta=\{\theta_{1},\ldots,\theta_{N}\}$ and $\Phi=\{\phi_{1},\ldots,\phi_{N}\}$. From condition (4.4), we get that $0<\vartheta<1$. Since all conditions in (4.2) hold, one can easily find that all the conditions of Lemma 4.5 are satisfied. It follows from Lemma 4.5 and (4.12) that

4.13 $$ \bigl(x_{1}^{n+1},x_{2}^{n+1}, \ldots,x_{N}^{n+1}\bigr)\rightarrow\bigl(x_{1}^{*},x_{2}^{*}, \ldots ,x_{N}^{*}\bigr),\quad \mbox{as }n\rightarrow\infty. $$

Using the definition of $\|\cdot\|_{*}$ and (4.12), one can easily show that

4.14 $$\begin{aligned}& \bigl\Vert \bigl(\bigl(x_{1}^{1},\ldots,x_{N}^{1} \bigr),\ldots,\bigl(x_{1}^{n},\ldots,x_{N}^{n} \bigr)\bigr)-\bigl(\bigl(x_{1}^{*},\ldots ,x_{N}^{*}\bigr),\ldots, \bigl(x_{1}^{*},\ldots,x_{N}^{*}\bigr)\bigr) \bigr\Vert _{*} \\& \quad = \sum_{j=1}^{n-1} \bigl\Vert \bigl(x_{1}^{j+1},\ldots,x_{N}^{j+1}\bigr)- \bigl(x_{1}^{*},\ldots,x_{N}^{*}\bigr) \bigr\Vert _{*}+ \bigl\Vert \bigl(x_{1}^{1},\ldots,x_{N}^{1} \bigr)-\bigl(x_{1}^{*},\ldots,x_{N}^{*}\bigr) \bigr\Vert _{*} \\& \quad \leq \sum_{j=1}^{n-1}\bigl(1- \alpha_{j}(1-\vartheta)\bigr) \bigl\Vert \bigl(x_{1}^{j},x_{2}^{j}, \ldots ,x_{N}^{j}\bigr)-\bigl(x_{1}^{*},x_{2}^{*}, \ldots,x_{N}^{*}\bigr) \bigr\Vert _{*} \\& \qquad {}+\sum_{j=1}^{n-1} \alpha_{j} \bigl\Vert \bigl(e_{1'}^{j}, \ldots,e_{N'}^{j}\bigr) \bigr\Vert _{*}+\sum _{j=1}^{n-1}\alpha_{j} \bigl\Vert \bigl(e_{1''}^{j},\ldots,e_{N''}^{j}\bigr) \bigr\Vert _{*} \\& \qquad {}+\sum_{j=1}^{n-1} \bigl\Vert \bigl(q_{1}^{j},\ldots,q_{N}^{j}\bigr) \bigr\Vert _{*}+\bigl(1-\alpha_{0}(1-\vartheta )\bigr) \bigl\Vert \bigl(x_{1}^{0},x_{2}^{0}, \ldots,x_{N}^{0}\bigr) \\& \qquad {}-\bigl(x_{1}^{*},x_{2}^{*},\ldots,x_{N}^{*} \bigr) \bigr\Vert _{*}+\alpha_{0} \bigl\Vert \bigl(e_{1'}^{0}, \ldots ,e_{N'}^{0}\bigr) \bigr\Vert _{*} \\& \qquad {}+ \bigl\Vert \bigl(e_{1''}^{0}, \ldots,e_{N''}^{0}\bigr) \bigr\Vert _{*}+ \bigl\Vert \bigl(q_{1}^{0},\ldots,q_{N}^{0}\bigr) \bigr\Vert _{*} \\& \quad = \sum_{j=0}^{n-1}\bigl(1- \alpha_{j}(1-\vartheta)\bigr) \bigl\Vert \bigl(x_{1}^{j},x_{2}^{j}, \ldots ,x_{N}^{j}\bigr)-\bigl(x_{1}^{*},x_{2}^{*}, \ldots,x_{N}^{*}\bigr) \bigr\Vert _{*} \\& \qquad {}+\sum_{j=0}^{n-1} \alpha_{j} \bigl\Vert \bigl(e_{1'}^{j}, \ldots,e_{N'}^{j}\bigr) \bigr\Vert _{*}+\sum _{j=0}^{n-1} \bigl\Vert \bigl(e_{1''}^{N}, \ldots,e_{N''}^{N}\bigr) \bigr\Vert _{*}+\sum _{j=0}^{n-1} \bigl\Vert \bigl(q_{1}^{j}, \ldots,q_{N}^{j}\bigr) \bigr\Vert _{*} \\& \quad = \sum_{j=0}^{n-1}\bigl\{ \bigl(1- \alpha_{j}(1-\vartheta)\bigr) \bigl\Vert \bigl(x_{1}^{j},x_{2}^{j}, \ldots ,x_{N}^{j}\bigr)-\bigl(x_{1}^{*},x_{2}^{*}, \ldots,x_{N}^{*}\bigr) \bigr\Vert _{*} \\& \qquad {}+\alpha_{j} \bigl\Vert \bigl(e_{1'}^{j}, \ldots,e_{N'}^{j}\bigr) \bigr\Vert _{*}+ \bigl\Vert \bigl(e_{1''}^{j},\ldots ,e_{N''}^{j}\bigr) \bigr\Vert _{*}+ \bigl\Vert \bigl(q_{1}^{j}, \ldots,q_{N}^{j}\bigr) \bigr\Vert _{*}\bigr\} . \end{aligned}$$

It follows from (4.13) and (4.14) that

$$\bigl\Vert \bigl(\bigl(x_{1}^{1},\ldots,x_{N}^{1} \bigr),\ldots,\bigl(x_{1}^{n},\ldots,x_{N}^{n} \bigr)\bigr)-\bigl(\bigl(x_{1}^{*},\ldots ,x_{N}^{*}\bigr),\ldots, \bigl(x_{1}^{*},\ldots,x_{N}^{*}\bigr)\bigr) \bigr\Vert _{*} \rightarrow0, \quad \mbox{as }n\rightarrow \infty. $$

Consequently, $\{x_{i}^{n}\}_{n=0}^{\infty}$ ($i=1,\ldots,N$) are Cauchy sequences in *H*. Hence, there exists $x_{i}^{*}\ (i=1,\ldots,N)\in{H}$ such that $x_{i}^{n}\rightarrow{x_{i}^{*}}\ (i=1,\ldots,N)\in{H}$ as $n\rightarrow\infty$. This completes the proof. □

### Remark

Equation (4.4) is the key condition for the convergence analysis in our algorithm. Moreover, the problem which we are solving has *n* elements. So, we consider the special case for one element. If we choose $\theta_{1}=\frac{1}{2}$, $\delta=2$, $\lambda_{1}=6.5$, $\kappa_{1}=1$, $\pi_{1}=1$, $\mu_{1}=1$, it follows that $\rho_{1}\in(\frac{5+\sqrt{7}}{8},\frac{5-\sqrt{7}}{8})$, which implies that the algorithm introduced in our paper is feasible.
